# Neuro desensitization of trigger points using interferential current with different amplitude modulated frequencies: A randomized controlled trial

**DOI:** 10.1097/MD.0000000000042405

**Published:** 2025-05-09

**Authors:** Hisham M. Hussein, Ahmed A. Ibrahim, Mohammad A. Aloraifi, Dina S. Abotaleb, Mohamed S. Ali, Monira I. Aldhahi, Aisha Ansari, Alaa Samir

**Affiliations:** a Department of Basic Sciences for Physical Therapy, Faculty of Physical Therapy, Cairo University, Giza, Egypt; b Department of Physical Therapy, College of Applied Medical Sciences, University of Ha’il, Ha’il, Saudi Arabia; c Department of Physical Therapy, King Khalid Hospital, Ha’il, Saudi Arabia; d Department of Rehabilitation Sciences, College of Health and Rehabilitation Sciences, Princess Nourah bint Abdulrahman University, Riyadh, Saudi Arabia.

**Keywords:** electrotherapy, pain, parameters, physiotherapy, sleep

## Abstract

**Background:**

To compare the effectiveness of 3 different amplitude modulated frequency (AMFs) of interferential current (IFC) on pain, range of motion (ROM), and function in subjects with chronic trigger points (TrPs) of the upper trapezius muscle.

**Methods:**

One hundred twenty subjects, 78 (65%) females and 42 (35%) males completed this study. All of them had latent TrPs in the upper fibers of the trapezius. They were randomly assigned into 4 groups; 3 experimental groups received IFC with different AMFs (4, 80, and 130 Hz) plus standard manual treatment while the 4th group (control group) received the standard treatment only. Pain intensity, cervical lateral flexion ROM, and function were assessed pre- and post-4 weeks of intervention. Average pain episodes and sleeping quality were assessed throughout the 3 months preceding and those following the intervention.

**Results:**

Post-intervention measures demonstrated statistical improvement in all outcomes interventions compared to the baseline (*P* ≤ .02). Pain, ROM, and function were equally improved in the 3 experimental groups compared to the control (*P* ≤ .04). 4 Hz group showed statistically significant difference compared to the 80 Hz group (*P* = .002) regarding pain, and statistically significant difference compared to 130 Hz and control group regarding the average pain episodes (*P* = .003 and.002, respectively). The 4 Hz group demonstrated a statistically significant difference compared to all other groups (*P* < .001) in Favor of the sleeping quality.

**Conclusion:**

Adding IFC to manual techniques improves pain, ROM, function, average pain episodes, and sleep quality in subjects with upper trapezius TrPs. 4 Hz AMF seems to have superior effects in terms of pain and average pain episodes compared to 80 and 100 Hz AMFs.

## 1. Introduction

Interferential current (IFC) is a medium frequency (1000–10,000 Hz) alternating current. In which 2 slightly outphased medium-frequency currents are used to produce a low-frequency effect.^[1]^ This effect is solely attributed to the difference in frequency (Amplitude modulated frequency [AMF]) between the 2 medium frequency currents. It has been hypothesized that the AMFs between 1 and 200 Hz were suitable for muscular contraction and desensitization of the nervous system and decreased pain experience.^[2]^

As it exhibits the advantageous features of facing less skin impedance, deep penetration, and high levels of comfort compared to other pain-modulating currents,^[3]^ IFC is commonly used in treating musculoskeletal pain and injuries.^[1]^ It has been used previously in the treatment of chronic muscular pain and myofascial pain syndrome.^[1,4,5]^

Myofascial pain syndrome is a common form of chronic musculoskeletal pain that contributes to a significant financial burden and job-related disability.^[6]^ The presence of a palpable overexcited spot inside a tight band of skeletal muscle fiber, known as myofascial trigger points (TrPs), is the main characteristic feature of myofascial pain syndrome. TrPs are usually seen or observed in the upper fibers of the trapezius muscle.^[7]^

It has been argued that TrPs are caused by the disturbance of muscle blood supply, and consequently the reduction in oxygen and nutrient supply. Such changes in blood supply have been attributed to micro-injuries and excessive stresses on the musculoskeletal system as the person assumes bad posture and executes incorrect movement patterns.^[8]^

Myofascial TrPs typically result in tenderness on compression, and localized or referred pain.^[6]^ Additionally, it might lead to muscle weakness, limited and painful movement, decline in mental and physical well-being.^[8]^ Moreover, changes in proprioception and kinematics have been observed in subjects having TrPs in cervical muscles.^[9]^

The use of different AMFs has been derived from the principles of the use of TENS currents. However, direct investigation of different AMFs used with IFC is scarce. Previous studies were performed to investigate the analgesic effect of different AMF in healthy subjects.^[10,11]^ There were only 2 studies that were concerned with the analgesic effect of different AMFs in subjects with musculoskeletal pain. Gundog and colleagues^[12]^ compared the effects of 3 AMFs (80, 100, and 120 Hz) on pain, range of motion (ROM), and function in 60 patients diagnosed with knee osteoarthritis. Additionally, Almeida and colleagues^[13]^ compared 2 different AMFs (2 and 100 Hz) along with different carrier waves (2 and 4 KHz) on pain associated with low back dysfunction. Yet, Almeida and colleagues’ study consisted of a single treatment session and was interested in pain as the only outcome measure.

Neither of the previously mentioned studies investigated subjects having TrPs, both studies did not report the clinical significance of their results except for the numeric pain rating scale (NPRS), which was reported by Almeida and colleagues. The results of the 2 studies were contradicting where Gundog and colleagues reported similar effects of different AMFs. Almeida and colleagues found that 100 Hz AMF was better than 2 Hz.

This study aimed to compare the effectiveness of 3 different AMFs of IFC on pain, ROM, and function in subjects with chronic TrPs of the upper trapezius muscle.

## 2. Material and methods

### 2.1. Design

A triple-blind randomized controlled trial.

### 2.2. Setting

This study was conducted between May 25th and November 30th, 2024 in a local university clinic, in Ha’il, Saudi Arabia.

### 2.3. Ethical issues

The current study was approved by the University of Ha’il ethical committee (NO: H-2023-289), and the study was prospectively registered at the Clinical Trial Registry (NCT05892991). Its procedures were conducted as per the declaration of Helsinki Guidelines.^[14]^ The reporting guidelines for randomized clinical trials (CONSORT) were also followed. The subjects signed a consent form to participate before the start of the study.

### 2.4. Duration

Four weeks of intervention.

### 2.5. Assessment timeline

Pain, cervical active range of motion (AROM), and function were assessed before and immediately after the end of the intervention. The average number of pain attacks and insomnia severity were measured during the 3 months pre and after intervention (Table 1).

**Table 1:** Schedule of the data collection throughout the study.

Outcome measure/time of assessment	Before treatment	After treatment	3 months follow up
Pain	✓	✓	
Range of motion	✓	✓	
Function	✓	✓	
The average number of painful episodes	✓		✓
Sleep Quality	✓		✓

### 2.6. Participants

Subjects were recruited from local University students, employees, and teaching staff. The study was advertised via e-mail, social media announcements, written announcements, and verbal communications. These inclusion criteria were both sexes, 17 to 45 years old, unilateral pain affecting any of the cervical, neck, or shoulder, pain experience for >3 months, pain intensity 2 or more on the NPRS, having signs of chronic (latent) TrPs in the upper fibers of the trapezius.

Subjects with cervical and/or shoulder pathologies such as disc lesion, arthritis or compression problems, neurological symptoms in the upper extremity as radiculopathy, previous related surgery, athletic lifestyle, heavy occupation-related activities, trauma to the cervical spine (whiplash injury), osteoporosis, complex regional pain syndrome, thoracic outlet syndrome, and regular analgesic drugs were excluded.

### 2.7. Outcome measures

Initial, post-intervention, and follow-up assessments were conducted by an experienced physical therapist. At the initial visit, an interview with subjects was conducted to describe the purpose of the study and answer any inquiries. Further assessments were conducted after obtaining their consent to participate.

Demographic data (gender, age, weight, height, and BMI), related habits like positioning, usage of smartphones or computers, and sleeping routine were reported.

#### 2.7.1. Trigger point confirmation

Diagnosis of latent TrPs was performed with the participant in the supine position. Using a pincer grip, the therapist palpated the entire upper trapezius. The therapist searched for Simon criteria to determine the existence of TrPs.^[15]^ These criteria were (1) the presence of a taut band in the affected muscle; (2) the presence of a highly sensitive spot within the taut band; (3) Local twitch response, (4) a referred pain pattern that is the pain radiating to the posterolateral side of the neck, and/or mastoid process area, and/or the temporal bone, and/or the angle of the jaw). The examination was performed bilaterally. If the participant reported the previously mentioned symptoms, but they were not experienced on a regular daily basis, this point was considered chronic (latency) TrPs.^[8]^

#### 2.7.2. Pain intensity

The intensity of pain experienced at the time of initial assessment was assessed using the NPRS. NPRS is simply a transverse 10 cm line numbered from 0 to 10. 0 represents the least degree of pain while 10 represents the extreme pain. Subjects were asked to choose the number that represent their current pain. Other work has established the validity and reliability of NPRS in assessing musculoskeletal pain.^[16]^

#### 2.7.3. AROM for the cervical spine

AROM of neck lateral flexion (ipsilateral and contralateral) was measured using a valid and reliable universal plastic goniometer. Subjects assumed a sitting position with the head and neck in the neutral position. The fulcrum of the goniometer was placed on the spine of the 7th cervical vertebra, the fixed arm was placed vertically to the ground, and the movable arm was placed in the midline of the head and neck with its end directed to the vertex of the head. The subject was asked to bend his head sideway as much as possible then return to the neutral position and repeat for the opposite direction. The procedure was repeated 3 times, and the average value was used for analysis.

#### 2.7.4. Function

The functional level was determined using a validated Arabic version of the neck disability index (NDI).^[17]^ The NDI is described as a 10-item questionnaire with a score range from 0 to 50 points (0%–100%). Higher NDI scores indicate lower levels of functional abilities. The interpretation of the NDI score was as follows: 5 to 14 points (10%–28%) for mild disability, 15 to 24 points (30%–48%) for moderate disability, 25 to 38 points (50%–68%) for severe disability, and above 34 points (68%) for complete disability.^[18]^

#### 2.7.5. Number of episodes of muscular pain due to trigger point activation

This outcome was assessed through a direct question asking the participant to determine the average number of TrPs-related pain attacks during the last month.

#### 2.7.6. Sleep quality

Sleep quality was assessed using an Arabic version of the insomnia severity index (ISI). This scale has been developed to assess the quality of sleep and the severity of insomnia. It is a 7-item questionnaire that requires subjects to rate the quality of sleep and any related problem. It contains questions related to subjective qualities of sleep, the severity of symptoms, sleep patterns, the degree to which insomnia interferes with daily life, how noticeable insomnia is to surrounding people, and the level of distress caused by sleep problems. Subject’s responses range from 0 to 4, on a Likert-type scale where the higher scores refer to more acute symptoms of insomnia. ISI has been used in different clinical situations^[19,20]^ and demonstrated good reliability and validity.^[21]^

### 2.8. Interventions

The standard treatment that was administered to all groups is the INIT. This technique was performed per guidelines described in previous studies.^[22,23]^ The standard treatment was applied 3 times per week for 4 weeks.

#### 2.8.1. Positional release

The positional release technique was applied to the affected upper trapezius muscle fibers. Positional release technique was performed by compressing the predetermined TrPs with the simultaneous changing of the length of the muscle to a comfortable shortened position where the reported pain decreases by 50% or more (position of ease). Once the position of ease was reached, it was maintained for 30 seconds. This procedure was repeated for 3 times per session.^[8]^

#### 2.8.2. Muscle energy technique

The participant was instructed to simultaneously move the treated shoulder toward the ipsilateral ear and side-flex the head toward the shoulder against manual resistance provided by the therapist’s hand to produce an isometric contraction. This isometric contraction was held for 10 seconds. Then the participant was instructed to relax while the therapist performed contralateral side bending and ipsilateral rotation to elongate the fibers of the upper trapezius muscle. The stretch was held for 30 seconds, and the procedure was repeated 3 to 5 times per session.^[23]^

#### 2.8.3. Progressive pressure release

The therapist applied manual pressure through a pincer grasp over the TrP. The participant was instructed to report feedback when they experienced moderate but bearable pain corresponding to a level 7 out of 10 where 1 represents no pain and 10 indicates unbearable pain. The applied pressure was maintained until the pain intensity decreased to 3 out of 10. Then, the pressure was increased again till the pain intensity returned to 7. This technique was applied for 90 seconds.^[22,23]^

#### 2.8.4. Experimental treatment

Three out of the 4 groups received IFC with fixed parameters except for the AMF. Each experimental group was named after its specific AMF value as 130, 80, and 4 Hz groups. The IFC was applied using a Chattanooga device (Intellect© Advanced). The parameters were 4 KHz, 2 poles (premodulated mode), and the current intensity was determined by the strong but comfortable tingling sensation when the 130 and 80 Hz AMFs while the intensity was raised till visible twitch contractions with the 4 Hz AMF. Two – medium-sized – vacuum electrodes were placed at both sides of the trigger point. The duration of each session was 30 minutes which was repeated 3 times per week for 4 weeks.

### 2.9. Sample size calculation

The sample size was calculated using the pain intensity as the outcome of interest, using G*Power software (3.1.9.7; Heinrich-Heine-Universität Dusseldorf, Dusseldorf, Germany). Calculations were designed to determine a minimal clinical difference equal to 2 points – on NPRS – between groups.^[24]^ The significant level was.05, and the power was 80%. The estimated desired total sample size for the study was 120. To compensate for possible dropouts, 132 subjects were recruited (33 per group).

### 2.10. Concealment and allocation

Subjects were allocated to groups by a researcher not involved in assessment or treatment. Permuted blocks generated using https://www.randomization.com/ were used for the allocation to assure equal and random allocation. Randomization codes were kept confidential in sealed opaque envelopes and sequentially numbered to ensure concealed allocation.

### 2.11. Statistical design

SPSS software 23 (SPSS, Chicago, IL) was used to perform all analyses. Mean ± SD was used to express data. Kolmogorov–Smirnov test was used to assess normality of data. One-way analysis of variance (ANOVA) was used to compare the means of the 4 groups. Within groups, differences were assessed using paired *t*-tests. *P* < .05 was considered significant. Tukey test was used for post hoc analysis when necessary. The within-group effect size was calculated using Cohen *d* coefficient. An effect size >0.8 was considered large while 0.5 was moderate, and <0.2 indicated a small effect size.^[22]^ eta squared (η^2^) was used to assess the ANOVA effect size of the between-group differences.

## 3. Results

**Figure 1.**
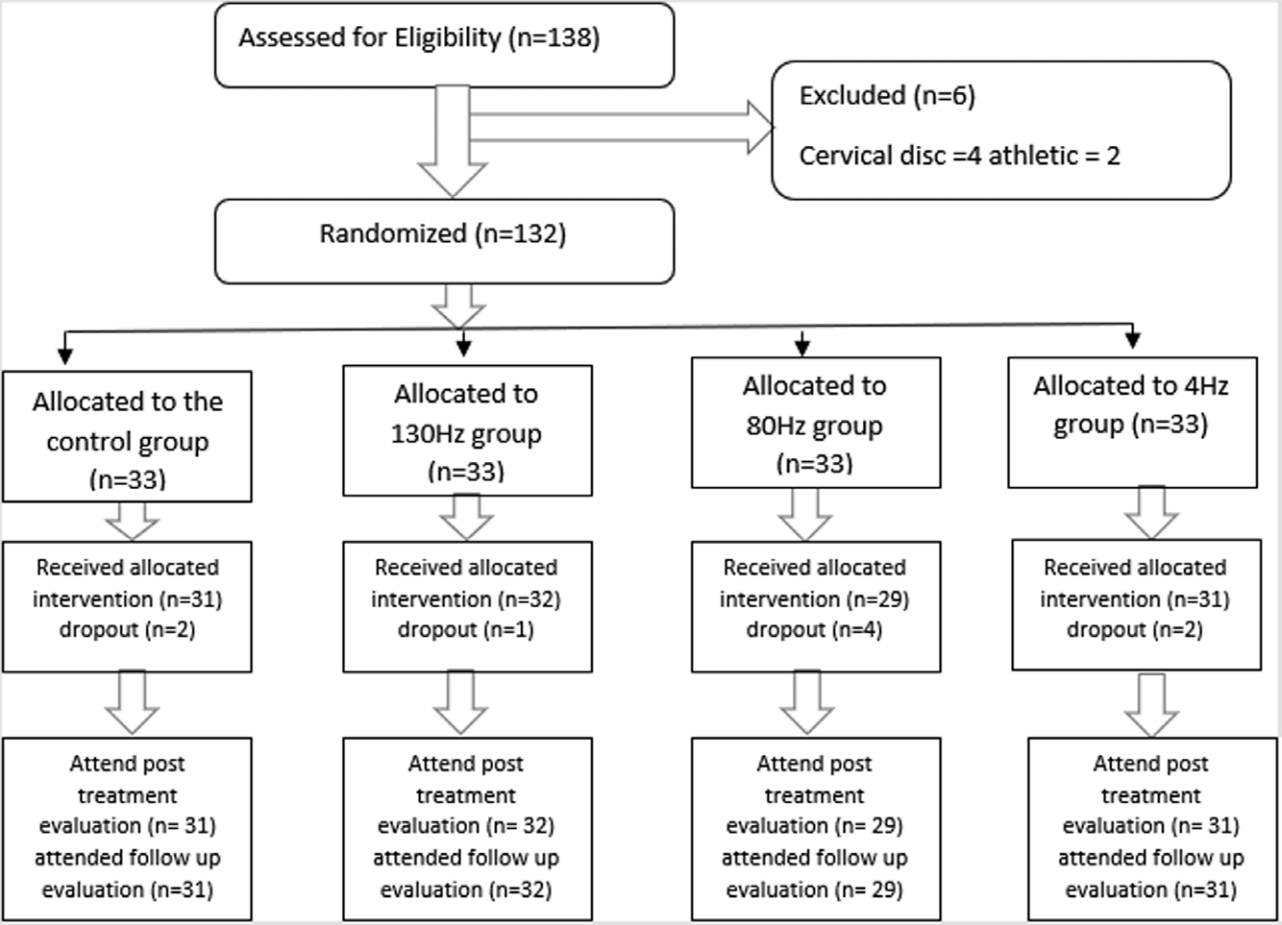
Flow chart.

A total of 120 subjects completed this study, of which 78 (65%) were females and 42 (35%) were males. The right side was affected in 73 (60.8 %) of the subjects while the left side was affected in the remaining 47 (39.2 %) subjects. 4 subjects were withdrawn due to the absence of 3 successive sessions, 3 other subjects had to move outside the city after joining the study, and 2 subjects were excluded due to personal reasons (Fig. 1).

Two subjects, 1 in the 130 Hz group and 1 in the 80 Hz group, reported mild irritation at the site of the electrode placement which was resolved when the proper electrode-skin contact was assured. The main characteristics of the subjects were similar in all groups (Table 2).

**Table 2:** Comparisons of the characteristics of the 4 groups.

	CG	130 Hz	80 Hz	4 Hz	F	*P*
Age	29.60 ± 9.42	29.30 ± 8.91	29.93 ± 9.35	30.40 ± 9.04	.080	.971
Weight	60.12 ± 6.50	61.15 ± 5.51	61.56 ± 6.30	61.50 ± 5.00	.382	.715
Height	1.64 ± 0.08	1.65 ± 0.07	1.64 ± 0.9	1.66 ± 0.08	.454	.766
BMI	26.49 ± 4.16	26.86 ± 4.79	27.18 ± 3.7	26.30 ± 4.15	.256	.857

CG = control group, F = Anova F value, *P* = significance.

### 3.1. One-way ANOVA results

The between groups analysis of the pretreatment data did not demonstrate statistical significance difference in favor of ipsilateral flexion (F = 1.245, DF = 3, *P* = .297), contralateral flexion (F = .459. DF = 3, *P* = .711), NPRS (F = 1.77, DF = 3, *P* = .156), and NDI (F = .069, DF = 3, *P* = 976). Similarly, the values of the average pain episodes (F = .292, DF = 3, *P* = .831) and ISI (F = .996, DF = 3, *P* = .398) during the 3 months before the intervention did not demonstrate any between-groups statistical differences.

The between-groups analysis of the posttreatment data demonstrated statistically significant differences in favor of ipsilateral lateral flexion (F = .292, DF = 3, *P* = .831. η^2^ =.16), contralateral lateral flexion (F = 7.721, DF = 3, *P* < .001, η^2^ =.24), NPRS(F = 15.658, DF = 3, *P* < .001, η^2^ =.28), NDI(F = 32.212, DF = 3, *P* < .001, η^2^ =.45), pain episode (F = 6.168, DF = 3, *P* < .001, η^2^ =.13), and ISI (F = 30.390, DF = 3, *P* < .001, η^2^ =.44).

The post hoc analysis using Tukey test revealed the following: (1) the ipsilateral lateral flexion of the 130 Hz group, 80 Hz group, and 4 Hz groups was statistically significantly different compared to the control group with *P* values equal < .001, .02, and .001, respectively. (2) The contralateral lateral flexion of the 130 Hz group, 80 Hz group, and 4 Hz group was statistically significantly different compared to the control group with *P* values equal < .001, .002, <.001, respectively. (3) The pain intensity in the 130 Hz group, 80 Hz group, and 4 Hz groups was statistically significantly different compared to the control group (*P* values equal < .001, .04, <.001, respectively). Meanwhile, the 4 Hz group was statistically significantly different compared to the 80 Hz group (*P* = .002) (Table 3). (4) The average pain episode during the 3 months posttreatment in the 4 Hz group was statistically significantly different compared to the control group (*P* = .002) and 130 Hz group (*P* = .003). (5) The ISI values in the 4 Hz groups were statistically significantly different compared to all other groups (*P* < .001). Meanwhile, these values in the 130 Hz group were statistically significantly different compared to the control group (*P* < .001) (Table 4).

**Table 3:** Within and between-groups analysis of the ROM, Pain, and function.

	Ipsilateral flexion				Contralateral flexion				Pain				NDI			
	Pre Mean ± SD	Post Mean ± SD	MD	*P*	Pre Mean ± SD	Post Mean ± SD	MD	*P*	Pre Mean ± SD	Post Mean ± SD	MD	*P*	Pre Mean ± SD	Post Mean ± SD	MD	*P*
Control group	23.36 ± 4.42	27.03 ± 6.25	3.66	<.001	25.36 ± 2.97	30.10 ± 3.90	4.73	<.001	4.73 ± 1.01	3.26 ± 1.20	1.46	<.001	45.56 ± 6.97	38.63 ± 6.7	6.93	.001
130 Hz group	22.36 ± 2.48	32.96 ± 4.07*	10.60	<.001	25.46 ± 2.41	35.03 ± 3.21*	9.56	<.001	4.56 ± 1.04	2.03 ± .71*	2.53	<.001	45.60 ± 7.16	26.16 ± 4.95*	19.44	<.001
80 Hz group	21.96 ± 2.42	30.93 ± 5.80*	8.96	<.001	25.96 ± 2.37	33.73 ± 4.20*	7.76	<.001	4.30 ± .95	2.63 ± .85*	1.66	<.001	44.86 ± 6.69	29.20 ± 6.27*	15.66	<.001
4 Hz group	22.10 ± 2.65	32.77 ± 4.26*	10.06	<.001	25.23 ± 2.57	35.40 ± 3.76*	10.16	<.001	4.86 ± 1.0	1.76 ± 0.85*†	3.10	<.001	45.36 ± 7.04	25.93 ± 4.74*	19.43	<.001

MD = mean difference, SD = standard deviation, *P* = significance.

* Significant compared to the control group.

† Significant compared to the 80 Hz group.

**Table 4:** Within and between-groups analysis of average of pain episode and insomnia disability index.

	Pain episodes				Insomnia severity index			
	Pre Mean ± SD	Post Mean ± SD	MD	*P*	Pre Mean ± SD	Post Mean ± SD	MD	*P*
Control group	2.86 ± .81	1.90 ± .80	.96	<.001	16.60 ± 4.27	14.13 ± 3.92	2.46	.001
130 Hz group	2.63 ± 1.09	1.86 ± .68	.76	.001	16.66 ± 4.19	10.53 ± 3.35*	6.13	<.001
80 Hz group	2.76 ± 1.04	1.50 ± .73	1.26	<.001	18.20 ± 4.75	12.65 ± 3.37	5.63	<.001
4 Hz group	2.80 ± .99	1.13 ± .93*†	1.66	<.001	16.56 ± 4.22	6.53 ± 2.16*†‡	10.03	<.001

SD = standard deviation, *P* = significance.

* Significant compared to the control group.

† Significant compared to 130 Hz group.

‡ Significant compared to 80 Hz group.

### 3.2. Within-group comparisons

There were statistically significant differences between the pretreatment and posttreatment values of all outcomes as demonstrated in Tables 3 and 4. The largest mean differences were observed between the CG and 130 Hz group in the ipsilateral lateral flexion (MD = 5.93°) group and between the control group and 4 Hz group in contralateral flexion (MD = 5.30°). Regarding pain intensity, the largest mean difference was observed between the CG and the 4 Hz group (MD = 1.5 cm). These 2 groups demonstrated the largest mean difference regarding the NDI score as well (MD = 12.70). Similarly, the mean differences between the 4 Hz group and the control group were the largest regarding the average of pain episodes (MD = .76), and ISI (MD = 7.6).

## 4. Discussion

This study used 3 AMFs of IFC on trapezius TrPs and assessed 5 outcomes namely, pain, ROM, function, average of pain episodes, and sleeping quality. The results of the 4 groups were better than the second assessment in favor of all outcomes. All AMFs were equally effective on pain and function where the values were better than the control group. The 4 Hz seems to have the best results on pain when compared to the 80 Hz and on ISI when compared to all other groups.

Several studies were conducted to investigate the therapeutic effects of IFC. However, a limited number of studies aimed to compare different parameters of the IFC. Almeida and colleagues studied the immediate analgesic effect of the IFC on patients with chronic low back pain. After using different carrier frequencies and different AMFs (2 and 100 Hz), they reported immediate improvement in pain level after using the 100 Hz AMF. This finding contradicts the current study where the 4 Hz AMF was better in terms of pain intensity and average pain episodes during the 3 months posttreatment. This difference could be attributed to the duration of the intervention where Almeida and colleagues performed a single session while the current study used a 4-week intervention program that consisted of standard physical therapy plus IFC.^[13]^ Additionally, the study conducted by Almeida and colleagues did not use a fixed carrier frequency, instead, a mix of carrier frequencies (2 and 4 KHz) and AMFs (2 and 100 Hz) were applied meanwhile, we used a single carrier frequency (4 Hz) in the current study.

In another study, Fuentes and colleagues investigated the analgesic effect of 2 AMFs namely 0 and 100 Hz. The sample consisted of 46 healthy subjects who were exposed to mechanically induced pain. Contrary to the current study, Fuentes did not find a statistical difference between using 0 and 100 Hz. Such findings were also reported in an earlier study when different AMFs (20, 60, 100, 140, 180, 220 Hz) were applied after inducing cold pain in a healthy subject. The outcomes of interest were pain intensity and pain unpleasantness in addition to pain threshold.^[11]^ Other researchers used different sets of AMFs (1, 10, and 100 Hz) on healthy subjects where the bipolar form of IFC using 4000 Hz carrier frequency was applied. This study reported no significant difference in the accommodation threshold. Meanwhile, there was a significant difference between the frequencies of 10 and 100 Hz, regarding the number of accommodations.^[25]^

The differences in the findings between the current study and those applied to healthy subjects are the nature of the problem where mechanically or cold-induced pain was artificially produced while subjects with TPs joined the current study. The second difference was the outcome measures, in the current study pain intensity and average episodes of pain were assessed but in previous work, the pressure pain threshold was assessed,^[10,11]^ pain intensity, and pain unpleasantness.^[10]^

Additionally, different AMFs were compared by Gundog and colleagues on patients having knee osteoarthritis. The outcome of interest in this study was pain, ROM, and function in addition to other measures such as pain medication and self-reported treatment effectiveness. Gundog and colleagues compared active IFC with 40, 100, and 180 Hz AMFs and sham IFC. The results demonstrated equal effectiveness of all AMFs especially which contradict the findings of the current study. The reasons for the contradiction might be attributed to the variation in the study population and the values of the AMFs used by Gundog and colleagues and those used in the current study.^[12]^

Correa and colleagues compared the effects of using different carrier frequencies of IFC namely 1 and 4 KHz on different outcomes like pain and function in patients with low back pain. Outcomes were assessed before, after, and at 4 months follow-up.^[26]^ Unlike the current findings, Correa reported no difference between the IFC and the placebo control in pain reduction. However, the IFC demonstrated less need for analgesic medications. The difference between this study and ours is that Correa study used different carrier frequencies and single AMF while in the current study, we used single carrier frequency and variable AMFs.

The current study findings can be appertained to several mechanisms. The use of electrical modalities can result in a desensitization of the nervous system by 2 common mechanisms, blocking the gate at the spinal level or releasing the endogenous opiates from the higher centers.^[3]^ The blocking of the gait is characterized by immediate effect but unfortunately, it is short lasting while the release of endogenous opiates has longer lasting effect. Using IFC with AMFs with 4 Hz has been suggested to stimulate the endogenous opiate mechanism, which might explain its superior effect on pain and pain episodes in comparison with the other AMFs which mainly work through the blocking of the gate at the spinal level. Previous work reported improvement in blood circulation following the application of IFC in different pathological cases.^[27,28]^ Improved blood supply is one of the important needs for desensitization of TrPs and consequently improving pain, and cervical ROM and might influence function and quality of sleep.^[27]^

The psychological and mental factors might also contribute to the beneficial findings associated with the application of IFC. Previous work showed that even the placebo application of IFC was associated with positive effects on pain. When the patient expects an analgesic effect from a certain treatment, this might improve the pain perception even if no active treatment was given.^[5]^ For example, 2 previous studies that compared active IFC to placebo control reported positive findings in the control group.^[29,30]^ This finding was reported in a third study conducted by Almeida and colleagues.^[5]^

Previous literature has limited research on the relationship between IFC and sleep quality. For example, Moretti and colleagues compared the use of combined therapy (IFC + ultrasound) with different treatment frequencies on different parameters including post-sleep inventory. Their results suggested better improvement in sleep quality after the application of both treatment frequencies. However, the AMFs of the IFC were not one of the study’s factors.^[31]^ Sleep quality is one of the important side effects that might be a result or a causative factor for the development of TrPs. So, improving sleeping quality could be crucial in improving the physical and mental well-being of the patients.

Future research should implement more studies on the different parameters of IFC including the AMFs. Also, addressing different types of TrPs such as active versus latent or central versus attachment could be beneficial. The authors encourage future researchers to incorporate sleeping quality in the mid and long term as a primary outcome measure where its response to the IFC and its correlation with the development of TrPs in the cervical region.

In light of the current results, incorporating IFC with different AMFs into the standard manual techniques could yield more effective results of treatment of TrPs in the upper trapezius muscle, especially in terms of pain, ROM, function, pain episodes, and sleeping quality. Using lower limits of the AMFs such as 4 Hz could be more promising in treating pain and reduction of pain episodes after the end of the interventions.

The limitations of the current study are that the results cannot be applied to other types or locations of the TrPs. No follow-up was measured for pain, ROM, and function was implemented. So, future research needs to consider this. Due to technical limitations, we could not assess the pressure pain threshold.

## 5. Conclusion

Adding IFC to manual techniques improves pain, ROM, function, average pain episodes, and sleep quality in subjects with upper trapezius TrPs. 4 Hz AMF seems to have superior effects in terms of pain and average pain episodes compared to 80 and 100 Hz AMFs.

## Acknowledgments

We would like to thank Princess Nourah bint Abdulrahman University for supporting this project through Princess Nourah bint Abdulrahman University Researchers Supporting Project number (PNURSP2025R 286), Princess Nourah bint Abdulrahman University, Riyadh, Saudi Arabia.

## Author contributions

**Conceptualization:** Hisham M. Hussein, Ahmed A. Ibrahim, Mohamed S. Ali, Monira I. Aldhahi, Alaa Samir.

**Data curation:** Hisham M. Hussein, Mohammad A. Aloraifi, Dina S. Abotaleb, Mohamed S. Ali, Aisha Ansari.

**Formal analysis:** Ahmed A. Ibrahim, Mohammad A. Aloraifi, Mohamed S. Ali, Monira I. Aldhahi, Aisha Ansari.

**Funding acquisition:** Monira I. Aldhahi.

**Investigation:** Ahmed A. Ibrahim, Dina S. Abotaleb, Aisha Ansari.

**Methodology:** Hisham M. Hussein, Mohammad A. Aloraifi, Dina S. Abotaleb, Mohamed S. Ali, Aisha Ansari.

**Project administration:** Mohammad A. Aloraifi.

**Resources:** Ahmed A. Ibrahim, Mohammad A. Aloraifi, Dina S. Abotaleb, Mohamed S. Ali, Monira I. Aldhahi, Alaa Samir.

**Supervision:** Hisham M. Hussein.

**Software:** Monira I. Aldhahi, Alaa Samir.

**Validation:** Alaa Samir.

**Writing – original draft:** Hisham M. Hussein, Monira I. Aldhahi, Aisha Ansari, Alaa Samir.

**Writing – review & editing:** Hisham M. Hussein, Ahmed A. Ibrahim, Monira I. Aldhahi, Alaa Samir.
